# Supplementary material for: Subcellular spatial transcriptomics identifies three mechanistically different classes of localizing RNAs
Source: Nat Commun. 2022 Oct 26;13:6355. doi: 10.1038/s41467-022-34004-2 (PMC9606379; doi:10.1038/s41467-022-34004-2)
Supplement: Supplementary file 1 — Supplementary Information [file 41467_2022_34004_MOESM1_ESM.pdf]

Subcellular spatial transcriptomics identifies three mechanistically different classes of localizing RNAs

SUPPLEMENTARY INFORMATION

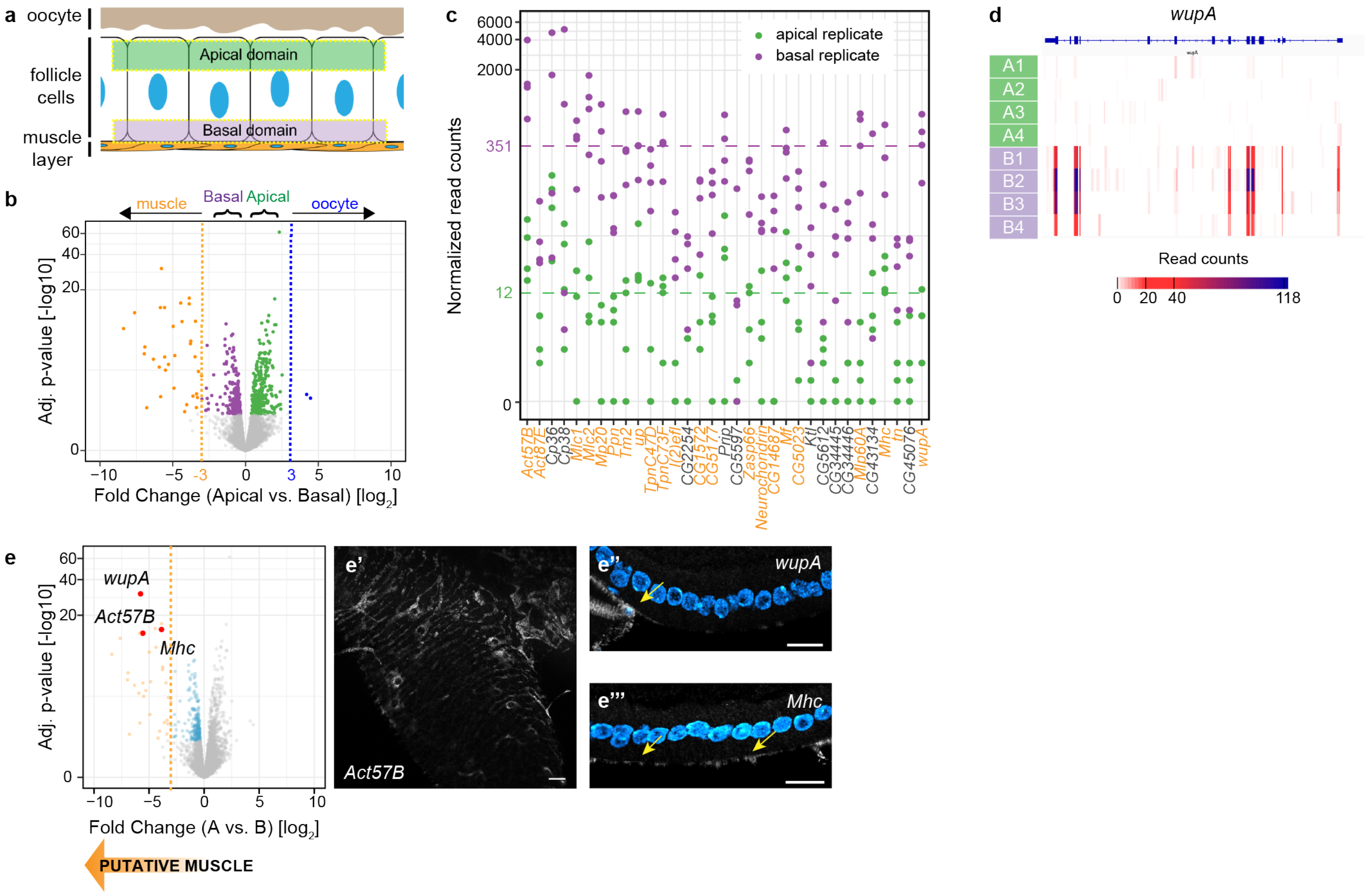
a
oocyte
follicle cells
muscle layer
Apical domain
Basal domain
b
muscle
Basal
Apical
oocyte
Adj. p-value [-log10]
Fold Change (Apical vs. Basal) [log2]
c
Normalized read counts
apical replicate
basal replicate
351
12
Act57B
Mhc
wupA
d
wupA
A1
A2
A3
A4
B1
B2
B3
B4
Read counts
0
20
40
118
e
wupA
Act57B
Mhc
Adj. p-value [-log10]
Fold Change (A vs. B) [log2]
PUTATIVE MUSCLE
e'
Act57B
e''
wupA
e'''
Mhc

Supplementary Figure 1. Identification of LCM contaminant RNAs. a) Spatial organization of the FE and surrounding tissues. The FE is bounded by the oocyte on its apical side and a thin layer of circular muscle fibers on the basal side. b) Volcano plot depicting apical vs. basal log_2-transformed fold change (apical vs. basal) values (x axis) by negative log_{10}-transformed adjusted p-value (y axis) for all genes analyzed by RNA-seq. Grey points represent genes below the significance threshold, with FDR adj-p value ≥ 0.1. Significantly enriched genes that exhibit a log2FC < - 3 are highlighted in orange and represent putative muscle contaminants (n=33). Significantly enriched genes that exhibit a log2FC > 3 are highlighted in blue and represent putative oocyte contaminants (n=2). Green points and purple points represent *bona fide* apical and basal RNAs respectively (FDR adj-p value < 0.1, -3 ≤ log2FC ≤ 3). c) Number of reads mapping to significantly enriched genes with log2FC < -3 (basal contaminants) on log scale. The low mean read count in the apical fragments (green points, avg = 12) with respect with the basal fragments (purple points, avg= 351) is an indication of contamination of the basal fragments from neighboring tissues and results in the generation of high |log2FC| values. Genes annotated as expressed or having a function in muscle tissue (FlyBase) are highlighted in orange. d) Integrative Genomic Viewer (IGV) representation of reads mapping to the gene model of the basal contaminant *wupA*, with the heatmap showing the number of mapped reads in each apical (n=4, A1-A4) or basal (n=4, B1-B4) sample. e) smFISH validation of *Act57B* (e'), *wupA* (e''), and *Mhc* (e''') RNAs among putative basal contaminants (orange dots, log2FC < -3 and FDR adj-p value <0.1). Arrows indicate expression of each RNA predominantly in the muscle tissue. The circular fiber organization surrounding each egg chamber can be appreciated by the expression of *Act57B* (top view, e'). FDR adjusted p-values in panels b and e were estimated by Wald test with the DESeq() function within the DESeq2 R package. Nuclei (cyan) are stained with DAPI. Scale bars 10 µm. Source data are provided as a Source Data file.

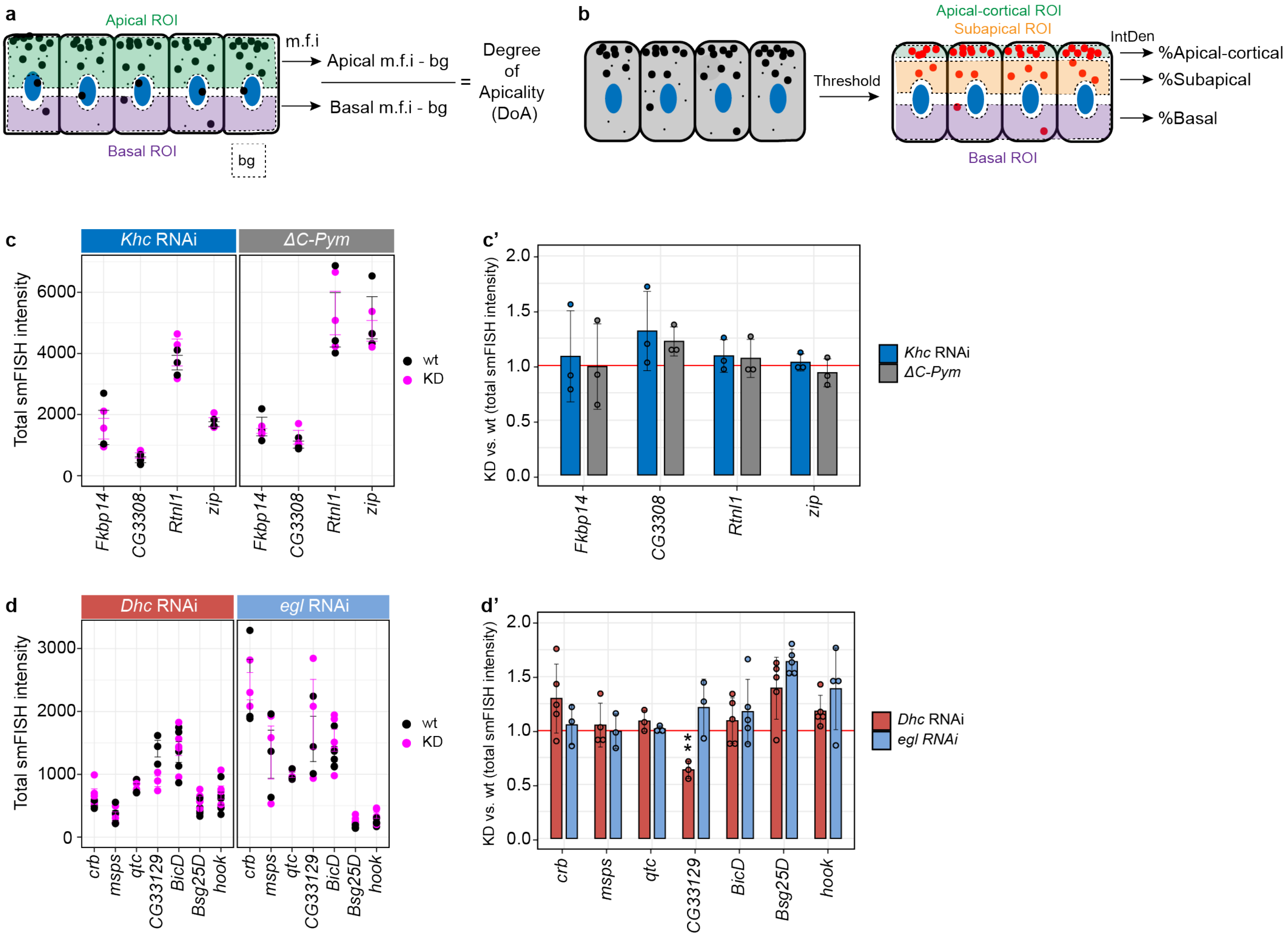

a
Apical ROI
m.f.i
Apical m.f.i - bg
Basal m.f.i - bg
=
Degree of Apicality (DoA)
Basal ROI
bg
b
Threshold
Apical-cortical ROI
Subapical ROI
IntDen
%Apical-cortical
%Subapical
%Basal
Basal ROI
c
Khc RNAi
ΔC-Pym
Total smFISH intensity
wt
KD
Fkbp14
CG3308
Rtnl1
zip
c'
KD vs. wt (total smFISH intensity)
Khc RNAi
ΔC-Pym
d
Dhc RNAi
egl RNAi
Total smFISH intensity
wt
KD
crb
msps
qtc
CG33129
BicD
Bsg25D
hook
d'
KD vs. wt (total smFISH intensity)
Dhc RNAi
egl RNAi
**

Supplementary Figure 2. smFISH signal quantification. a-b) Schematic representation of how signal quantification was performed to measure changes in RNA localization in this study. a) To measure the Degree of Apicality (DoA), adjacent wild-type (wt) or knock-down (KD) FC were segmented into apical and basal ROIs encompassing the apical and basal cytoplasm, respectively. The nuclear and peri-nuclear areas were excluded from measurements, as these regions often contain bright transcriptional foci. The DoA values in wt or KD cells were calculated as the ratio between the apical and basal background(bg)-subtracted mean fluorescent intensity (m.f.i). b) To quantify changes in the localization of adaptor RNAs upon treatment with translational inhibitors, smFISH signal threshold was first adjusted to increase signal-to-noise ratio and the integrated signal density (IntDen) measured in the apical-cortical, subapical, and basal ROIs that were drawn as depicted in the cartoon. c-d) Total (apical + basal) smFISH signal quantification (arb.units) (mean ± s.e.m) of RNAs analyzed in this study in KD (magenta) and wt (black) cells in *Khc* RNAi and *ΔC-Pym* mosaic tissues (c) and *Dhc* RNAi and *egl* RNAi mosaic tissues (d). The mean KD/wt(total smFISH intensity) value (± 2SD) for each RNA in each condition (c'-d') was tested against a null hypothesis H_0 of KD/wt=1 (red horizontal line), corresponding to no change in total signal intensity between mutant and wild-type cells (one-sided t-test, mu=1, alternative="less"). Asterisks indicate mean values that are significantly lower than the reference value of mu=1 (*=p<0.05; **=p<0.01; ***=p<0.001; at least n=3 biologically independent replicates were analyzed in each condition). With the exception of *CG33129* RNA, which displayed significant RNA degradation in *Dhc* RNAi cells compared to wt cells (p= 0.007956), apical RNA amount did not decrease under both RNAi conditions. Note that, when imaging the lowly expressed *Bsg25D* and *hook,* the high laser power required to image ATTO-633-labeled smFISH probes resulted in mCherry signal bleed-through that added up to the the total smFISH signal of mCherry-marked mutant clones, resulting in slightly higher smFISH intensity values in RNAi cells compared to unmarked wild-type cells. Source data are provided as a Source Data file.

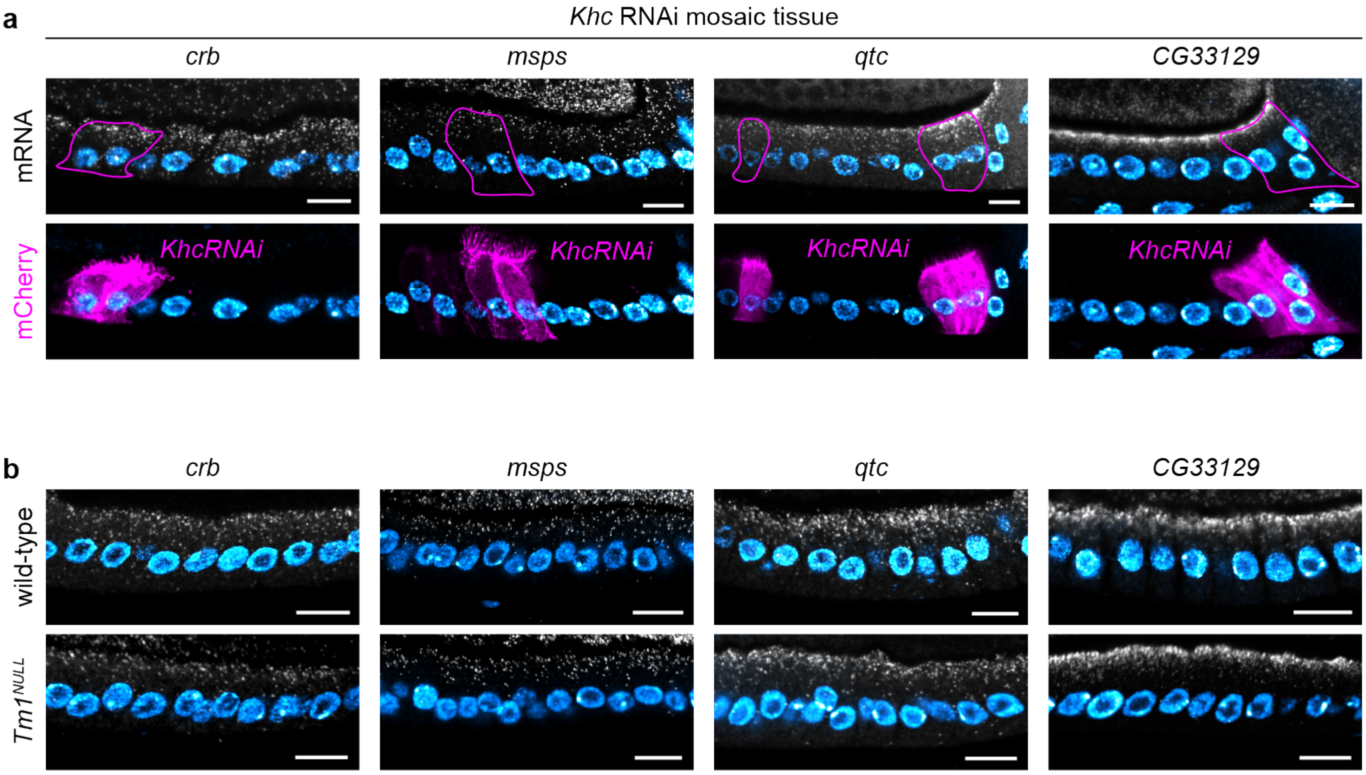
a
Khc RNAi mosaic tissue
crb
msps
qtc
CG33129
mRNA
mCherry
KhcRNAi
KhcRNAi
KhcRNAi
KhcRNAi
b
crb
msps
qtc
CG33129
wild-type
Tm1NULL

Supplementary Figure 3. Effects of kinesin-1 complex disruption on apical RNAs. a) Localization of apical RNAs by smFISH in *Khc* RNAi mosaic tissue. Mutant cells are marked by the expression of CD8-mCherry (lower panels) and highlighted with a continuous line in smFISH images (upper panels). Neighboring wild-type cells are unmarked. b) Localization of apical RNAs by smFISH in wild-type and $Tm1^{NULL}$ egg chambers. Nuclei (cyan) are stained with DAPI. Scale bars 10 µm.

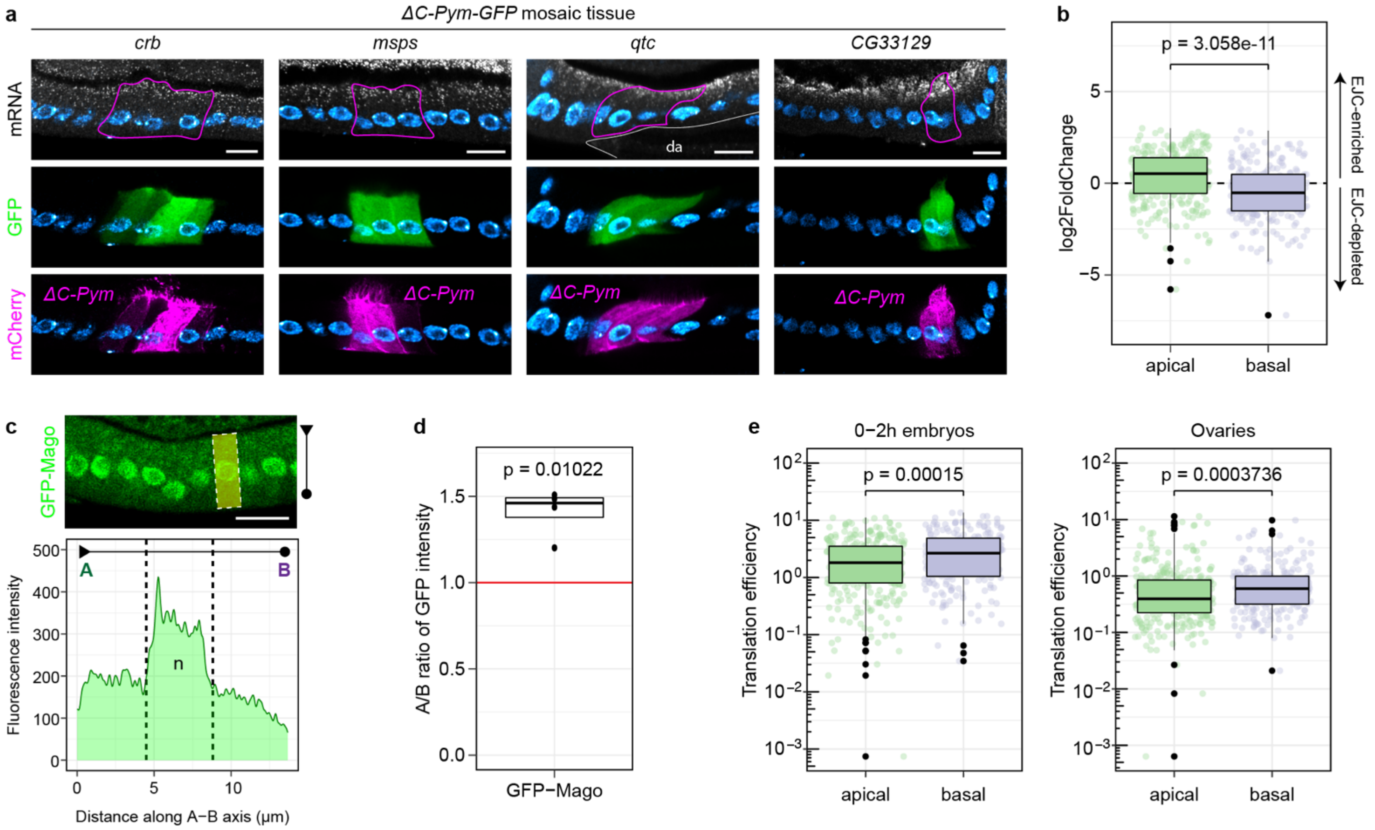
a
ΔC-Pym-GFP mosaic tissue
crb
msps
qtc
CG33129
mRNA
GFP
mCherry
ΔC-Pym
da
b
p = 3.058e-11
log2FoldChange
EJC-enriched
EJC-depleted
apical
basal
c
GFP-Mago
Fluorescence intensity
A
B
n
Distance along A−B axis (μm)
d
p = 0.01022
A/B ratio of GFP intensity
GFP−Mago
e
0−2h embryos
p = 0.00015
Translation efficiency
apical
basal
Ovaries
p = 0.0003736
Translation efficiency
apical
basal

Supplementary Figure 4. Effects of EJC displacement on apical RNAs and analysis of EJC association with apical and basal RNAs. a) Localization of apical RNAs by smFISH in *ΔC-Pym-GFP* mosaic tissue. CD8-mCherry marks mutant cells (lower panels), highlighted with a continuous line in smFISH images (upper panels). GFP marks the expression of ΔC-Pym (middle panels). Neighboring wild-type cells are unmarked. Nuclei (cyan) are stained with DAPI. da = dorsal appendage. b) EJC enrichment estimates from Obrdlik et al.[1] of apical (green) and basal (purple) RNAs. EJC-enriched (log2FC>0) and EJC-depleted (RBP-enriched, log2FC<0) RNAs were subset as apical or basal according to our RNA-seq results, and their relative EJC-enrichment (log2FC, y axis) was analyzed. A dashed line indicates log2FC=0. Highlighted p-value has been estimated by two-sided Wilcoxon rank sum test (apical: n=288; basal: n=214). c) GFP-Mago expression in stage 10 FE (upper panel) and signal quantification along the A-B axis of the representative FC highlighted in yellow. Apical (triangle) to basal (circle) directionality is indicated above the x-axis. Two dashed lines delimit nuclear signal (n). d) Mean ratio between apical and basal GFP-Mago intensity. Values>1 indicate apical signal enrichment. A continuous red line at A/B=1 indicates the null hypothesis of equal distribution along the A-B axis. Highlighted p-value has been estimated by one-sample two-sided t-test (mu=1, n=4). e) Translation efficiency (log scale) of apical (green) and basal (purple) RNAs from published ribo-seq datasets. Left panel: 0-2h embryos[2]; right panel: control RNAi ovaries[3]. Translation efficiency (y-axis) was calculated as the ratio between the RNA ribosome density (TPM) measured by ribosome profiling and the RNA abundance (TPM) measured by RNA-seq from each study. RNAs were subset as apical or basal according to our RNA-seq results and their translation efficiency was analyzed. Highlighted p-values have been estimated by two-sided Wilcoxon rank sum test. 0-2h embryos, apical: n=297; 0-2h embryos, basal: n=207; ovaries, apical: n=300; ovaries, basal: n=213. Boxplots of panels b, d, e, show the median (horizontal line), the first and third quartiles (bounds of box), ± 1.5 × interquartile range (whiskers). Black dots represent outliers in panels b,e. Scale bars 10 µm. Source data are provided as a Source Data file.

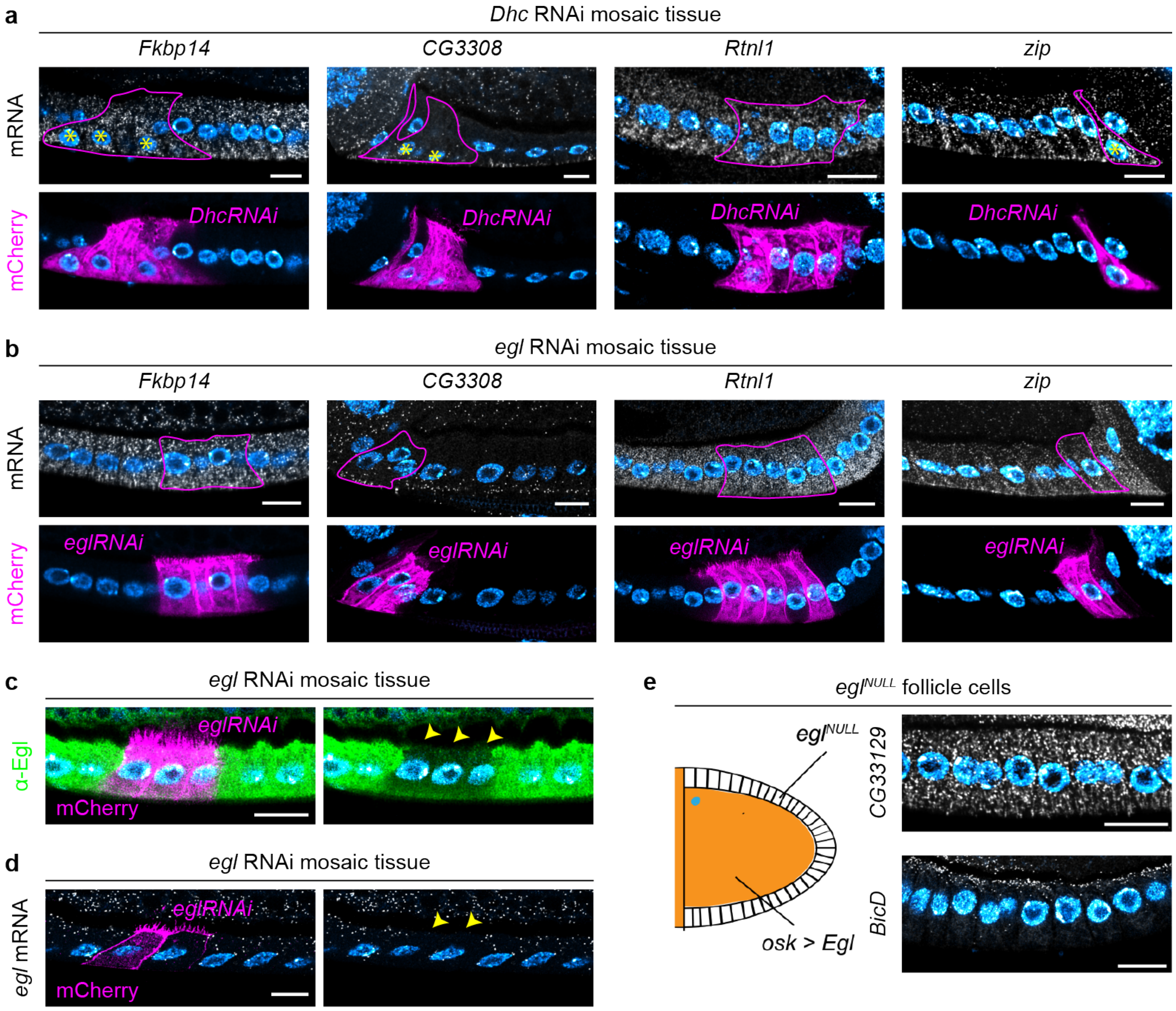
a
Dhc RNAi mosaic tissue
Fkbp14
CG3308
Rtnl1
zip
mRNA
mCherry
DhcRNAi
b
egl RNAi mosaic tissue
eglRNAi
c
egl RNAi mosaic tissue
α-Egl
mCherry
d
egl mRNA
e
egl^NULL follicle cells
egl^NULL
osk > Egl
CG33129
BicD

Supplementary Figure 5. Effects of dynein/BicD/Egl complex disruption on localizing RNAs. a-b) Localization of basal RNAs by smFISH in *Dhc* RNAi (a) and *egl* RNAi (b) mosaic tissue. Mutant cells are marked by the expression of CD8-mCherry (lower panels) and highlighted with a continuous line in smFISH images (upper panels). Neighboring wild-type cells are unmarked. Asterisks (*) indicate basal mispositioning of nuclei due to *Dhc* RNAi. c) Expression of Egl protein (green) in mosaic tissue containing wild-type (unmarked) and *eglRNAi* follicle cells (magenta) visualized by immunostaining. Arrowheads indicate the decrease of Egl signal in *eglRNAi* cells. d) Expression of *egl* RNA in a mosaic follicular epithelium containing wild-type (unmarked) and *eglRNAi* cells (magenta) visualized by smFISH using antisense *egl* probes. Arrowheads indicate depletion of *egl* RNA in *eglRNAi* cells. e) egl^{NULL} egg chambers in which the expression of Egl was rescued only in the germline, resulting in egl^{NULL} follicle cells ($egl^{NULL}FC$, see Materials and Methods for full genotype). In $egl^{NULL}FC$, *BicD* RNA was still apically enriched. In contrast, *CG33129* RNA was unlocalized, phenocopying *egl* RNAi condition (see Fig. 4b). Nuclei (cyan) are stained with DAPI. Scale bars 10 µm.

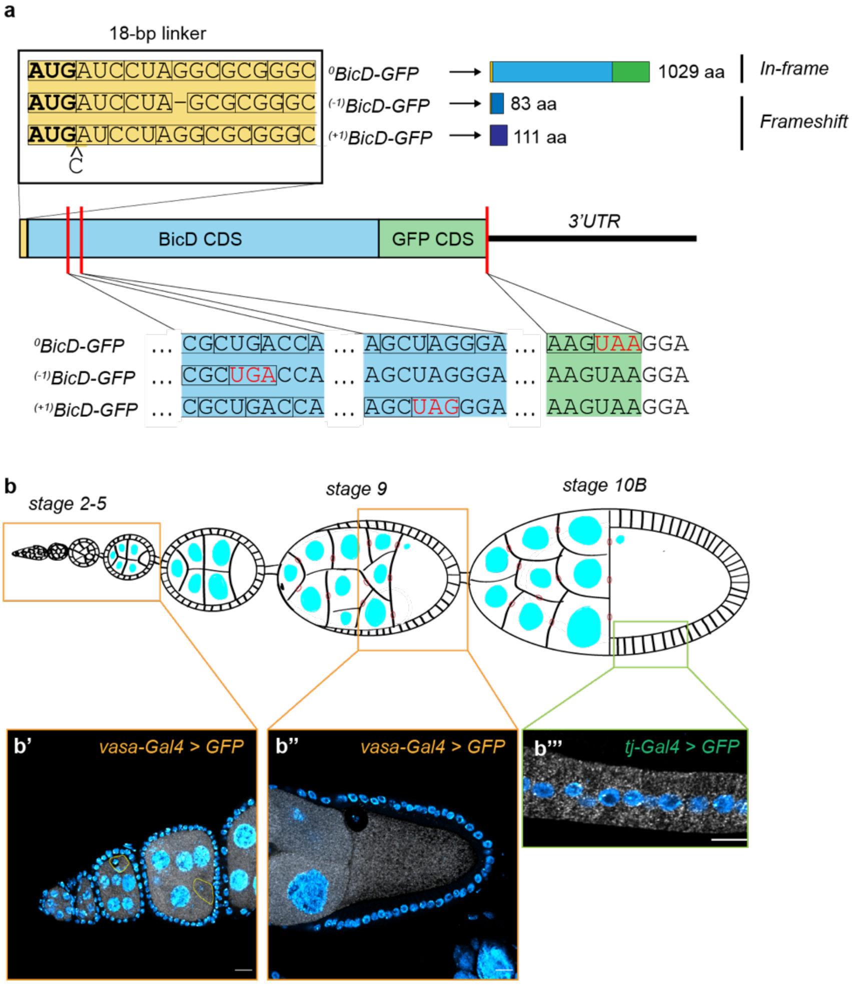
a
18-bp linker
AUGAUCCUAGGCGCGGGC
AUGAUCCUA-GCGCGGGC
AUGAUCCUAGGCGCGGGC
C
0BicD-GFP
(-1)BicD-GFP
(+1)BicD-GFP
1029 aa
83 aa
111 aa
In-frame
Frameshift
BicD CDS
GFP CDS
3'UTR
0BicD-GFP ... CGCUGACCA ... AGCUAGGGA ... AAGUAAGGA
(-1)BicD-GFP ... CGCUGACCA ... AGCUAGGGA ... AAGUAAGGA
(+1)BicD-GFP ... CGCUGACCA ... AGCUAGGGA ... AAGUAAGGA
b
stage 2-5
stage 9
stage 10B
b' vasa-Gal4 > GFP
b'' vasa-Gal4 > GFP
b''' tj-Gal4 > GFP

Supplementary Figure 6. BicD-GFP and GFP constructs. a) An 18-bp linker was placed upstream of full-length *BicD* fused in frame with *GFP* to generate frameshift mutations, in an effort to avoid the emergence of RNA localization phenotypes due to the disruption of *BicD* RNA sequence. Each construct differs only in the presence or absence of a single-nucleotide frameshift mutation in the 18-bp N-terminal linker (yellow box). In ^{0}*BicD-GFP* RNA, the N-terminal linker is translated in frame with *BicD* and *GFP* ORFs and produces a 1029 aa protein that contains a full-length BicD-GFP protein. In $^{(-1)}$*BicD-GFP* RNA, a G was deleted at position 10, causing a -1 frameshift and resulting in a 83 aa peptide. In $^{(+1)}$*BicD-GFP* RNA, a C was added in position 4, causing a +1 frameshift and resulting in a 111 aa peptide. Red lines indicate the predicted termination codons. b) Visualization by smFISH of *GFP* RNA (grayscale), carrying the same 3'UTR as BicD-GFP constructs in the early germline cysts (b'), in mid-stage oocyte (b'') and in the follicular epithelium (b'''). Germline expression was driven by *vasa-Gal4*; expression in the FE was driven by *traffic jam (tj)-Gal4*. Oocytes are highlighted in orange in b'. Apical is on the top, posterior is on the right. Nuclei (cyan) are stained with DAPI. Scale bars 10 µm.

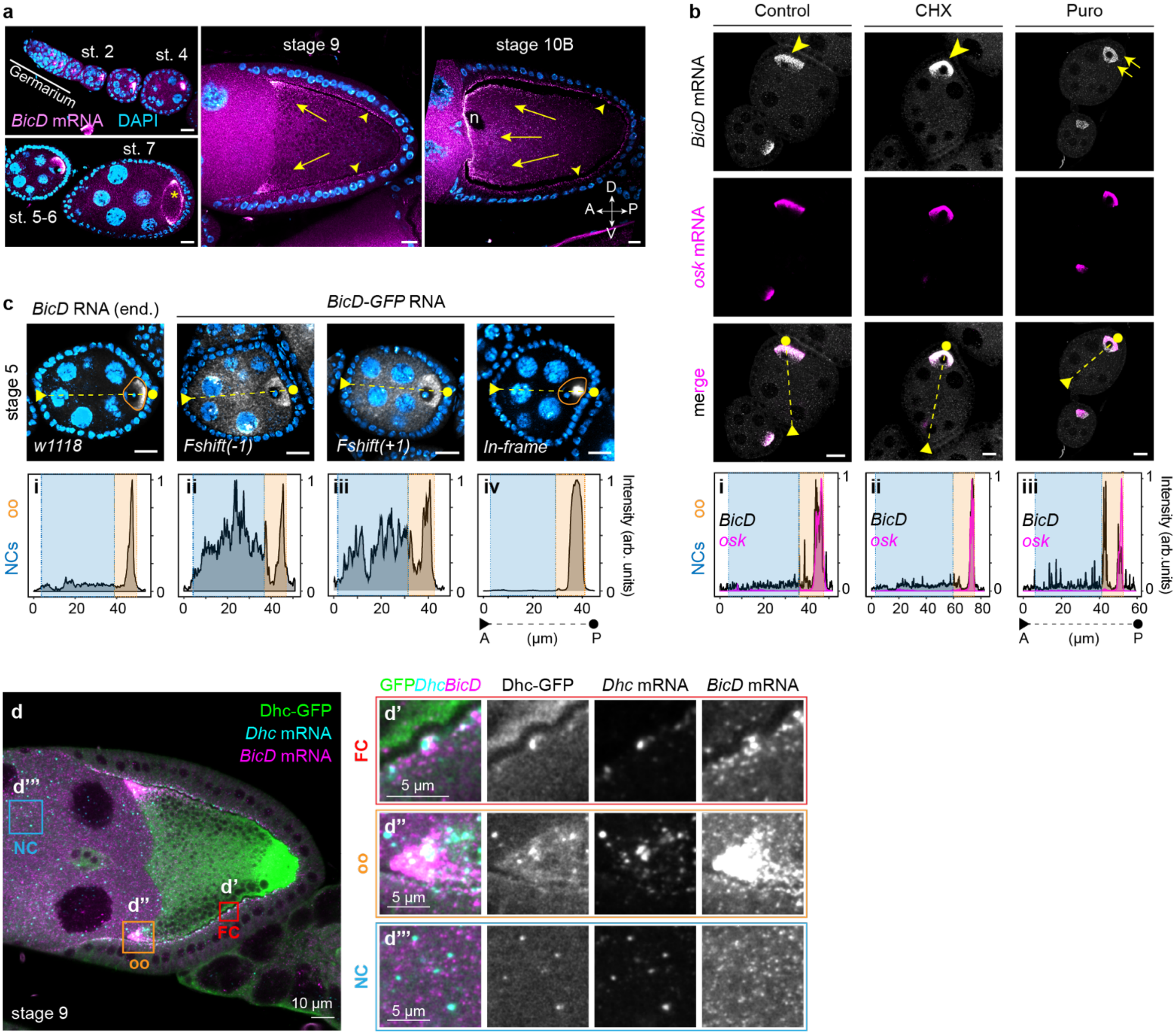
a
st. 2
st. 4
Germarium
BicD mRNA DAPI
st. 7
st. 5-6
stage 9
stage 10B
n
D
A
P
V
b
Control
CHX
Puro
BicD mRNA
osk mRNA
merge
i
ii
iii
BicD
osk
oo
NCs
Intensity (arb.units)
A
(µm)
P
c
BicD RNA (end.)
BicD-GFP RNA
stage 5
w1118
Fshift(-1)
Fshift(+1)
In-frame
iv
Intensity (arb. units)
d
Dhc-GFP
Dhc mRNA
BicD mRNA
d'''
NC
d''
d'
FC
oo
stage 9
10 µm
GFPDhcBicD
5 µm

Supplementary Figure 7. Translation-dependent and -independent mechanisms of *BicD* RNA localization in the germline. a) *BicD* RNA localization throughout oogenesis. *BicD* RNA is enriched in the two pro-oocytes in germarial region 2b, and becomes restricted to the oocyte from stage 1 onwards. At stage 5-6 *BicD* accumulates at the oocyte posterior. Upon MT rearrangement at stage 7 (*), *BicD* localizes at the oocyte corners, and begins to localize apically in FCs. At stage 9, *BicD* localizes at the anterior corners of the oocyte (arrows) and apically in columnar FCs (arrowheads). At stage 10, *BicD* localizes at the anterior and lateral cortex of the oocyte (arrows) and appears tightly apical in the columnar FCs (arrowheads). n = oocyte nucleus. A = anterior; P = posterior; D = dorsal; V = ventral. b) Stage 6 egg chambers in control, CHX or Puro conditions. In control and CHX conditions, *BicD* (arrowheads) co-localizes with *osk* at the oocyte posterior. Upon Puro treatment, only *BicD* RNA becomes ubiquitous in the oocyte (arrows). Intensity plots (lower panels) represent *BicD* (black) and *osk* (magenta) smFISH signal measured along the A-P axis in Control (i), CHX (ii) and Puro (iii) conditions. The anterior (triangle) to posterior (circle) cross-section along which each fluorescence signal was measured (arb. units) is shown. Note the maintenance of *BicD* RNA enrichment in the oocyte in all conditions. c) Localization of different *BicD* RNA species in stage 5 egg chambers. Intensity plots represent endogenous *BicD* (i), *Frameshift* (ii, iii) or *In-frame* (iv) BicD-GFP smFISH signal measured along the egg chamber A-P axis. Note the ubiquitous distribution of *Frameshift* RNA in the oocyte compared to the posterior localization of endogenous *BicD* or *In-frame* BicD-GFP RNA. Oocytes are highlighted in orange in *w1118* and *In-frame* panels. d) Localization of *BicD* RNA, *Dhc* RNA, and endogenously tagged Dhc-GFP in stage 9 egg chamber in FC apical cortex (d'), oocyte anterior corners (d''), and nurse cells (d'''). oo = oocyte; FC = follicle cell; NC = nurse cell. a = anterior; p = posterior. In a) and c) nuclei (cyan) are stained with DAPI. Scale bars 10 μm. Source data are provided as a Source Data file.

SUPPLEMENTARY REFERENCES

1. Obrdlik, A.; Lin, G.; Haberman, N.; Ule, J.; Ephrussi, A. The Transcriptome-Wide Landscape and Modalities of EJC Binding in Adult Drosophila. *Cell Rep.* **2019**, *28* (5), 1219-1236.e11. https://doi.org/10.1016/j.celrep.2019.06.088.

2. Greenblatt, E. J.; Obniski, R.; Mical, C.; Spradling, A. C. Prolonged Ovarian Storage of Mature Drosophila Oocytes Dramatically Increases Meiotic Spindle Instability. *Elife* **2019**, *8*. https://doi.org/10.7554/eLife.49455.

3. Greenblatt, E. J.; Spradling, A. C. Fragile X Mental Retardation 1 Gene Enhances the Translation of Large Autism-Related Proteins. *Science* **2018**, *361* (6403), 709–712. https://doi.org/10.1126/science.aas9963.
