# Supplementary material for: Subcellular spatial transcriptomics identifies three mechanistically different classes of localizing RNAs
Source: Nat Commun. 2022 Oct 26;13:6355. doi: 10.1038/s41467-022-34004-2 (PMC9606379; doi:10.1038/s41467-022-34004-2)
Supplement: Supplementary file 3 — Description of Additional Supplementary Files [file 41467_2022_34004_MOESM3_ESM.pdf]

Description of Additional Supplementary Files

File Name: Supplementary Data 1
Description: List of significantly enriched (FDR < 0.1) RNAs in apical and basal LCM samples. Apically enriched RNAs (n=306, sheet 1) are characterized by a positive log2FC (log2FC > 0) and are divided into “apical (bona fide)” (0 < log2FC ≤ 3) or “oocyte contaminant” (log2FC > 3). Basally enriched RNAs (n=249, sheet 2) are characterized by a negative log2FC (log2FC < 0) and have been divided into “basal (bona fide)” (-3 ≤ log2FC < 0) or “muscle contaminant” (log2FC < -3). Candidate RNAs chosen for validation by smFISH are highlighted in yellow. Sheet 3 contains the raw output of DEseq2 differential gene expression analysis, including genes below the significance threshold. The last column (“sig”) has been added to keep track of bona fide localizing RNAs (adj-p < 0.1), putative contaminants (sig_apical_contaminant/sig_basal_contaminant), and non-significant RNAs (Not sig).

File Name: Supplementary Data 2
Description: Sequence and annotations of BicD-GFP constructs generated in this study.

File Name: Supplementary Data 3
Description: List of smFISH oligos used in this study. Each column contains oligo sequences for all smFISH probe sets used in this study.

File Name: Supplementary Movie 1
Description: Example LCM of basal and apical fragments of a stage 10 follicular epithelium.
