# Supplementary material for: Subcellular spatial transcriptomics identifies three mechanistically different classes of localizing RNAs
Source: Nat Commun. 2022 Oct 26;13:6355. doi: 10.1038/s41467-022-34004-2 (PMC9606379; doi:10.1038/s41467-022-34004-2)
Supplement: Supplementary file 5 — Supplementary Data 2 [file 41467_2022_34004_MOESM5_ESM.pdf]

Supplementary Data 2

Sequences of BicD-GFP constructs

Legend:
CAAAATG Drosophila Kozak sequence
NNNN 18-bp linker
NNNN BicD CDS (excluding ATG and STOP)
NNNN GFP CDS (excluding ATG)
NNN predicted STOP codon
_ 1 nt deletion (-1 frameshift)
N 1 nt insertion (+1 frameshift)
NNNN predicted translated ORF

>^{0}BicD-GFP
CAAA**ATGATCCTAGGCGCGGCTCCAGCGCCAGCAACAACGGCCCATCGGCGGACCAATCCG**
TGCAAGACCTGCAAATGGAGGTGGAGCGCCTCACGCGAACTGGACCAGGTGTCCTCCGCC
AGCGCCCAGTCCGCCCAGTACGGACTGTCCCTGCTGGAGGAGAAGTCCGCCCTGCAGCAGAA
GTGCGAGGAACTGGAGACGCTCTACGACAACACGCGCCACGAACTGGACATCACACAGGAGG
CGCTGACCAAGTTTCAAACCTCACAGAAAGTGACCAACAAGACGGGCATCGAGCAGGAGGAC
GCTCTGCTGAACGAATCCGCAGCTAGGGAGACATCGCTCAACCTCCAGATATTTGATCTGGA
GAACGAGCTTAAGCAACTGCGCCATGAGTTGGAAAGGGTTCGCAATGAGCGCGATAGGATGC
TGCAGGAGAACTCGGATTTTGGGCGGGACAAGAGCGACAGCGAGGCGGATCGCCTACGCCTC
AAGTCCGAGCTGAAGGACCTTAAGTTCCGGGAGACGCGTATGCTTAGCGAATACTCGGAGCT
GGAGGAGGAGAACATATCGCTGCAAAAGCAGGTCTCCAGCCTGCGCAGCTCACAGGTGGAAT
TTGAAGGTGCCAAACACGAGATCCGTCGTCTCACTGAAGAAGTTGAGCTGTTGAATCAACAG
GTCGATGAGCTCGCCAATCTTAAAAAAATTGCCGAAAAACAAATGGAGGAAGCGCTAGAGAC
CTTACAGGGTGAACGTGAGGCGAAATATGCATTGAAGAAGGAACTGGATGGCCACTTGAACC
GTGAGTCTATGTACCACATCAGCAACCTCGCCTACAGCATACGCAGCAACATGGAAGACAAC
GCCAGCAACAACTCGGACGGTGAGGAGGAAAATCTGGCTCTTAAGCGTCTGGAGGCTGACCT
GAGCACCGAACTTAAATCTCCTGACGGCACCAAATGTGATCTCTTTTCGGAGATTCATCTGA
ACGAACTAAAGAAACTGGAGAAGCAGTTGGAGAGCATGGAAAGTGAGAAGACTCATCTGACG
GCGAATTTAAGGGAAGCTCAGACGAGTCTGGACAAGTCACAAAACGAGCTGCAGAACTTTAT
GTCTCGTCTGGCTCTTCTTGCGGCCCATGTCGATGCTCTAGTCCAGCTAAAGAAGCAGATCG
ATGTGAAGGAGCAGGGCAAGGAAGGTGGCCAGAAGAAGGATGAACTGGAGCAGCAGCTGCGA
GCGTTAATCTCGCAGTACGCCAACTGGTTTACGCTCTCCGCCAAGGAGATCGATGGCCTTAA
GACTGACATTGCTGAACTTCAGAAGGGACTCAACTATACGGACGCCACCACTACGCTGCGCA
ACGAGGTGACCAACCTTAAGAACAAGCTTCTTGCTACGGAACAAAAGTCACTGGACCTGCAG
AGCGATGTTCAAACTCTTACGCACATCTCGCAAAACGCTGGCCAAAGTCTGGGCTCAGCTCG
CAGTACATTGGTGGCCTTAAGCGACGATCTGGCGCAGCTGTATCACCTAGTTTGCACAGTCA
ACGGAGAGACACCGACGCGTGTTCTGCTCGATCATAAGACCGATGACATGAGCTTCGAAAAC
GATTCTTTGACTGCCATCCAGTCGCAATTCAAATCGGATGTCTTTATTGCCAAGCCCCAGAT
CGTCGAGGATCTGCAAGGGTTGGCGGATTCCGTGGAAATTAAGAAGTACGTGGATACAGTCA
GTGATCAGATCAAGTATCTGAAGACGGCTGTTGAGCATACCATTGATATGAATAAACACAAA
ATCCGCTCCGAGGGTGGCGACGCACTGGAGAAGGTTAACACAGAGGAGATGGAGGAACTGCA
GGAGCAGATAGTCAAGTTGAAGAGTTTGCTGTCCGTGAAGCGCGAGCAGATTGGAACTCTGC
GCAACGTGCTCAAGTCAAACAAGCAAACCGCTGAGGTGGCACTGACCAATCTCAAGTCCAAG
TATGAGAACGAGAAGATCATTGTCAGCGACACCATGTCCAAGCTACGTAATGAGCTCAGGCT
TCTTAAGGAGGATGCTGCCACATTCTCAAGCCTGCGTGCCATGTTCGCCGCTCGATGCGAGG
AGTATGTGACCCAGGTGGACGATCTCAACCGCCAATTGGAGGCAGCAGAGGAGGAGAAAAAG
ACTCTAAATCAGCTGTTGCGCTTGGCTGTCCAGCAGAAGCTGGCACTCACTCAGCGACTCGA

GGAGATGGAAATGGACCGCGAAATGCGTCACGTCCGTCGGCCGATGCCAGCCCAGCGTGGCA
CGAGCGGCAAGTCCTCCTTCAGCACGAGACCTTCGAGCAGGAATCCAGCAAGCAGTAACGCC
AATCCATTCGGCGGCCGCGTGAGCAAGGGCGAGGAGCTGTTCACCGGGGTGGTGCCCATCCT
GGTCGAGCTGGACGGCGACGTAAACGGCCACAAGTTCAGCGTGTCCGGCGAGGGCGAGGGCG
ATGCCACCTACGGCAAGCTGACCCTGAAGTTCATCTGCACCACCGGCAAGCTGCCCGTGCCC
TGGCCCACCCTCGTGACCACCCTGACCTACGGCGTGCAGTGCTTCAGCCGCTACCCCGACCA
CATGAAGCAGCACGACTTCTTCAAGTCCGCCATGCCCGAAGGCTACGTCCAGGAGCGCACCA
TCTTCTTCAAGGACGACGGCAACTACAAGACCCGCGCCGAGGTGAAGTTCGAGGGCGACACC
CTGGTGAACCGCATCGAGCTGAAGGGCATCGACTTCAAGGAGGACGGCAACATCCTGGGGCA
CAAGCTGGAGTACAACTACAACAGCCACAACGTCTATATCATGGCCGACAAGCAGAAGAACG
GCATCAAGGTGAACTTCAAGATCCGCCACAACATCGAGGACGGCAGCGTGCAGCTCGCCGAC
CACTACCAGCAGAACACCCCCATCGGCGACGGCCCCGTGCTGCTGCCCGACAACCACTACCT
GAGCACCCAGTCCGCCCTGAGCAAAGACCCCAACGAGAAGCGCGATCACATGGTCCTGCTGG
AGTTCGTGACCGCCGCCGGGATCACTCTCGGCATGGACGAGCTGTACAAGTAA

>$^{(-1)}$BicD-GFP

CAAAATGATCCTA_GCGCGGGCTCCAGCGCCAGCAACAACGGCCCATCGGCGGACCAATCCG
TGCAAGACCTGCAAATGGAGGTGGAGCGCCTCACGCGCGAACTGGACCAGGTGTCCTCCGCC
AGCGCCCAGTCCGCCCAGTACGGACTGTCCCTGCTGGAGGAGAAGTCCGCCCTGCAGCAGAA
GTGCGAGGAACTGGAGACGCTCTACGACAACACGCGCCACGAACTGGACATCACACAGGAGG
CGCTGACCAAGTTTCAAACCTCACAGAAAGTGACCAACAAGACGGGCATCGAGCAGGAGGAC
GCTCTGCTGAACGAATCCGCAGCTAGGGAGACATCGCTCAACCTCCAGATATTTGATCTGGA
GAACGAGCTTAAGCAACTGCGCCATGAGTTGGAAAGGGTTCGCAATGAGCGCGATAGGATGC
TGCAGGAGAACTCGGATTTTGGGCGGGACAAGAGCGACAGCGAGGCGGATCGCCTACGCCTC
AAGTCCGAGCTGAAGGACCTTAAGTTCCGGGAGACGCGTATGCTTAGCGAATACTCGGAGCT
GGAGGAGGAGAACATATCGCTGCAAAAGCAGGTCTCCAGCCTGCGCAGCTCACAGGTGGAAT
TTGAAGGTGCCAAACACGAGATCCGTCGTCTCACTGAAGAAGTTGAGCTGTTGAATCAACAG
GTCGATGAGCTCGCCAATCTTAAAAAAATTGCCGAAAAACAAATGGAGGAAGCGCTAGAGAC
CTTACAGGGTGAACGTGAGGCGAAATATGCATTGAAGAAGGAACTGGATGGCCACTTGAACC
GTGAGTCTATGTACCACATCAGCAACCTCGCCTACAGCATACGCAGCAACATGGAAGACAAC
GCCAGCAACAACTCGGACGGTGAGGAGGAAAATCTGGCTCTTAAGCGTCTGGAGGCTGACCT
GAGCACCGAACTTAAATCTCCTGACGGCACCAAATGTGATCTCTTTTCGGAGATTCATCTGA
ACGAACTAAAGAAACTGGAGAAGCAGTTGGAGAGCATGGAAAGTGAGAAGACTCATCTGACG
GCGAATTTAAGGGAAGCTCAGACGAGTCTGGACAAGTCACAAAACGAGCTGCAGAACTTTAT
GTCTCGTCTGGCTCTTCTTGCGGCCCATGTCGATGCTCTAGTCCAGCTAAAGAAGCAGATCG
ATGTGAAGGAGCAGGGCAAGGAAGGTGGCCAGAAGAAGGATGAACTGGAGCAGCAGCTGCGA
GCGTTAATCTCGCAGTACGCCAACTGGTTTACGCTCTCCGCCAAGGAGATCGATGGCCTTAA
GACTGACATTGCTGAACTTCAGAAGGGACTCAACTATACGGACGCCACCACTACGCTGCGCA
ACGAGGTGACCAACCTTAAGAACAAGCTTCTTGCTACGGAACAAAAGTCACTGGACCTGCAG
AGCGATGTTCAAACTCTTACGCACATCTCGCAAAACGCTGGCCAAAGTCTGGGCTCAGCTCG
CAGTACATTGGTGGCCTTAAGCGACGATCTGGCGCAGCTGTATCACCTAGTTTGCACAGTCA
ACGGAGAGACACCGACGCGTGTTCTGCTCGATCATAAGACCGATGACATGAGCTTCGAAAAC
GATTCTTTGACTGCCATCCAGTCGCAATTCAAATCGGATGTCTTTATTGCCAAGCCCCAGAT
CGTCGAGGATCTGCAAGGGTTGGCGGATTCCGTGGAAATTAAGAAGTACGTGGATACAGTCA
GTGATCAGATCAAGTATCTGAAGACGGCTGTTGAGCATACCATTGATATGAATAAACACAAA
ATCCGCTCCGAGGGTGGCGACGCACTGGAGAAGGTTAACACAGAGGAGATGGAGGAACTGCA
GGAGCAGATAGTCAAGTTGAAGAGTTTGCTGTCCGTGAAGCGCGAGCAGATTGGAACTCTGC
GCAACGTGCTCAAGTCAAACAAGCAAACCGCTGAGGTGGCACTGACCAATCTCAAGTCCAAG
TATGAGAACGAGAAGATCATTGTCAGCGACACCATGTCCAAGCTACGTAATGAGCTCAGGCT
TCTTAAGGAGGATGCTGCCACATTCTCAAGCCTGCGTGCCATGTTCGCCGCTCGATGCGAGG
AGTATGTGACCCAGGTGGACGATCTCAACCGCCAATTGGAGGCAGCAGAGGAGGAGAAAAAG
ACTCTAAATCAGCTGTTGCGCTTGGCTGTCCAGCAGAAGCTGGCACTCACTCAGCGACTCGA

GGAGATGGAAATGGACCGCGAAATGCGTCACGTCCGTCGGCCGATGCCAGCCCAGCGTGGCA
CGAGCGGCAAGTCCTCCTTCAGCACGAGACCTTCGAGCAGGAATCCAGCAAGCAGTAACGCC
AATCCATTCGGCGGCCGCGTGAGCAAGGGCGAGGAGCTGTTCACCGGGGTGGTGCCCATCCT
GGTCGAGCTGGACGGCGACGTAAACGGCCACAAGTTCAGCGTGTCCGGCGAGGGCGAGGGCG
ATGCCACCTACGGCAAGCTGACCCTGAAGTTCATCTGCACCACCGGCAAGCTGCCCGTGCCC
TGGCCCACCCTCGTGACCACCCTGACCTACGGCGTGCAGTGCTTCAGCCGCTACCCCGACCA
CATGAAGCAGCACGACTTCTTCAAGTCCGCCATGCCCGAAGGCTACGTCCAGGAGCGCACCA
TCTTCTTCAAGGACGACGGCAACTACAAGACCCGCGCCGAGGTGAAGTTCGAGGGCGACACC
CTGGTGAACCGCATCGAGCTGAAGGGCATCGACTTCAAGGAGGACGGCAACATCCTGGGGCA
CAAGCTGGAGTACAACTACAACAGCCACAACGTCTATATCATGGCCGACAAGCAGAAGAACG
GCATCAAGGTGAACTTCAAGATCCGCCACAACATCGAGGACGGCAGCGTGCAGCTCGCCGAC
CACTACCAGCAGAACACCCCCATCGGCGACGGCCCCGTGCTGCTGCCCGACAACCACTACCT
GAGCACCCAGTCCGCCCTGAGCAAAGACCCCAACGAGAAGCGCGATCACATGGTCCTGCTGG
AGTTCGTGACCGCCGCCGGGATCACTCTCGGCATGGACGAGCTGTACAAGTAA

>(+1)BicD-GFP
CAAA**ATGCATCCTAGGCGCGGCTCCAGCGCCAGCAACAACGGCCCATCGGCGGACCAATCC**
GTGCAAGACCTGCAAATGGAGGTGGAGCGCCTCACGCGCGAACTGGACCAGGTGTCCTCCGC
CAGCGCCCAGTCCGCCCAGTACGGACTGTCCCTGCTGGAGGAGAAGTCCGCCCTGCAGCAGA
AGTGCGAGGAACTGGAGACGCTCTACGACAACACGCGCCACGAACTGGACATCACACAGGAG
GCGCTGACCAAGTTTCAAACCTCACAGAAAGTGACCAACAAGACGGGCATCGAGCAGGAGGA
CGCTCTGCTGAACGAATCCGCAGCTAGGGAGACATCGCTCAACCTCCAGATATTTGATCTGG
AGAACGAGCTTAAGCAACTGCGCCATGAGTTGGAAAGGGTTCGCAATGAGCGCGATAGGATG
CTGCAGGAGAACTCGGATTTTGGGCGGGACAAGAGCGACAGCGAGGCGGATCGCCTACGCCT
CAAGTCCGAGCTGAAGGACCTTAAGTTCCGGGAGACGCGTATGCTTAGCGAATACTCGGAGC
TGGAGGAGGAGAACATATCGCTGCAAAAGCAGGTCTCCAGCCTGCGCAGCTCACAGGTGGAA
TTTGAAGGTGCCAAACACGAGATCCGTCGTCTCACTGAAGAAGTTGAGCTGTTGAATCAACA
GGTCGATGAGCTCGCCAATCTTAAAAAAATTGCCGAAAAACAAATGGAGGAAGCGCTAGAGA
CCTTACAGGGTGAACGTGAGGCGAAATATGCATTGAAGAAGGAACTGGATGGCCACTTGAAC
CGTGAGTCTATGTACCACATCAGCAACCTCGCCTACAGCATACGCAGCAACATGGAAGACAA
CGCCAGCAACAACTCGGACGGTGAGGAGGAAAATCTGGCTCTTAAGCGTCTGGAGGCTGACC
TGAGCACCGAACTTAAATCTCCTGACGGCACCAAATGTGATCTCTTTTCGGAGATTCATCTG
AACGAACTAAAGAAACTGGAGAAGCAGTTGGAGAGCATGGAAAGTGAGAAGACTCATCTGAC
GGCGAATTTAAGGGAAGCTCAGACGAGTCTGGACAAGTCACAAAACGAGCTGCAGAACTTTA
TGTCTCGTCTGGCTCTTCTTGCGGCCCATGTCGATGCTCTAGTCCAGCTAAAGAAGCAGATC
GATGTGAAGGAGCAGGGCAAGGAAGGTGGCCAGAAGAAGGATGAACTGGAGCAGCAGCTGCG
AGCGTTAATCTCGCAGTACGCCAACTGGTTTACGCTCTCCGCCAAGGAGATCGATGGCCTTA
AGACTGACATTGCTGAACTTCAGAAGGGACTCAACTATACGGACGCCACCACTACGCTGCGC
AACGAGGTGACCAACCTTAAGAACAAGCTTCTTGCTACGGAACAAAAGTCACTGGACCTGCA
GAGCGATGTTCAAACTCTTACGCACATCTCGCAAAACGCTGGCCAAAGTCTGGGCTCAGCTC
GCAGTACATTGGTGGCCTTAAGCGACGATCTGGCGCAGCTGTATCACCTAGTTTGCACAGTC
AACGGAGAGACACCGACGCGTGTTCTGCTCGATCATAAGACCGATGACATGAGCTTCGAAAA
CGATTCTTTGACTGCCATCCAGTCGCAATTCAAATCGGATGTCTTTATTGCCAAGCCCCAGA
TCGTCGAGGATCTGCAAGGGTTGGCGGATTCCGTGGAAATTAAGAAGTACGTGGATACAGTC
AGTGATCAGATCAAGTATCTGAAGACGGCTGTTGAGCATACCATTGATATGAATAAACACAA
AATCCGCTCCGAGGGTGGCGACGCACTGGAGAAGGTTAACACAGAGGAGATGGAGGAACTGC
AGGAGCAGATAGTCAAGTTGAAGAGTTTGCTGTCCGTGAAGCGCGAGCAGATTGGAACTCTG
CGCAACGTGCTCAAGTCAAACAAGCAAACCGCTGAGGTGGCACTGACCAATCTCAAGTCCAA
GTATGAGAACGAGAAGATCATTGTCAGCGACACCATGTCCAAGCTACGTAATGAGCTCAGGC
TTCTTAAGGAGGATGCTGCCACATTCTCAAGCCTGCGTGCCATGTTCGCCGCTCGATGCGAG
GAGTATGTGACCCAGGTGGACGATCTCAACCGCCAATTGGAGGCAGCAGAGGAGGAGAAAAA
GACTCTAAATCAGCTGTTGCGCTTGGCTGTCCAGCAGAAGCTGGCACTCACTCAGCGACTCG

AGGAGATGGAAATGGACCGCGAAATGCGTCACGTCCGTCGGCCGATGCCAGCCCAGCGTGGC
ACGAGCGGCAAGTCCTCCTTCAGCACGAGACCTTCGAGCAGGAATCCAGCAAGCAGTAACGC
CAATCCATTCGGCGGCCGCGTGAGCAAGGGCGAGGAGCTGTTCACCGGGGTGGTGCCCATCC
TGGTCGAGCTGGACGGCGACGTAAACGGCCACAAGTTCAGCGTGTCCGGCGAGGGCGAGGGC
GATGCCACCTACGGCAAGCTGACCCTGAAGTTCATCTGCACCACCGGCAAGCTGCCCGTGCC
CTGGCCCACCCTCGTGACCACCCTGACCTACGGCGTGCAGTGCTTCAGCCGCTACCCCGACC
ACATGAAGCAGCACGACTTCTTCAAGTCCGCCATGCCCGAAGGCTACGTCCAGGAGCGCACC
ATCTTCTTCAAGGACGACGGCAACTACAAGACCCGCGCCGAGGTGAAGTTCGAGGGCGACAC
CCTGGTGAACCGCATCGAGCTGAAGGGCATCGACTTCAAGGAGGACGGCAACATCCTGGGGC
ACAAGCTGGAGTACAACTACAACAGCCACAACGTCTATATCATGGCCGACAAGCAGAAGAAC
GGCATCAAGGTGAACTTCAAGATCCGCCACAACATCGAGGACGGCAGCGTGCAGCTCGCCGA
CCACTACCAGCAGAACACCCCCATCGGCGACGGCCCCGTGCTGCTGCCCGACAACCACTACC
TGAGCACCCAGTCCGCCCTGAGCAAAGACCCCAACGAGAAGCGCGATCACATGGTCCTGCTG
GAGTTCGTGACCGCCGCCGGGATCACTCTCGGCATGGACGAGCTGTACAAGTAA
