# Supplementary material for: Subcellular spatial transcriptomics identifies three mechanistically different classes of localizing RNAs
Source: Nat Commun. 2022 Oct 26;13:6355. doi: 10.1038/s41467-022-34004-2 (PMC9606379; doi:10.1038/s41467-022-34004-2)
Supplement: Supplementary file 8 — Reporting Summary [file 41467_2022_34004_MOESM8_ESM.pdf]

nature portfolio

Corresponding author(s): Dr. Anne Ephrussi
Last updated by author(s): 11 September 2022

Reporting Summary

Nature Portfolio wishes to improve the reproducibility of the work that we publish. This form provides structure for consistency and transparency in reporting. For further information on Nature Portfolio policies, see our Editorial Policies and the Editorial Policy Checklist.

Statistics

For all statistical analyses, confirm that the following items are present in the figure legend, table legend, main text, or Methods section.

n/a	Confirmed	
☐	☒	The exact sample size (*n*) for each experimental group/condition, given as a discrete number and unit of measurement
☐	☒	A statement on whether measurements were taken from distinct samples or whether the same sample was measured repeatedly
☐	☒	The statistical test(s) used AND whether they are one- or two-sided<br>*Only common tests should be described solely by name; describe more complex techniques in the Methods section.*
☐	☒	A description of all covariates tested
☐	☒	A description of any assumptions or corrections, such as tests of normality and adjustment for multiple comparisons
☐	☒	A full description of the statistical parameters including central tendency (e.g. means) or other basic estimates (e.g. regression coefficient) AND variation (e.g. standard deviation) or associated estimates of uncertainty (e.g. confidence intervals)
☐	☒	For null hypothesis testing, the test statistic (e.g. *F*, *t*, *r*) with confidence intervals, effect sizes, degrees of freedom and *P* value noted<br>*Give P values as exact values whenever suitable.*
☒	☐	For Bayesian analysis, information on the choice of priors and Markov chain Monte Carlo settings
☒	☐	For hierarchical and complex designs, identification of the appropriate level for tests and full reporting of outcomes
☒	☐	Estimates of effect sizes (e.g. Cohen's *d*, Pearson's *r*), indicating how they were calculated

Our web collection on statistics for biologists contains articles on many of the points above.

Software and code

Policy information about availability of computer code

Data collection	smFISH image collection: Leica Application Suite X (LAS X) on a Leica Sp8-CSU confocal microscope. Functional annotation of Drosophila genes: data collected from Flybase (release FB2020_6).
Data analysis	RNA-seq raw reads were processed with Galaxy. The quality of reads was checked with FastQC (v. 0.69) after each processing step. Raw reads were trimmed with Trimmomatic (v. 0.36.4) to remove adapter sequences and filtered from rRNA sequences with SortMeRNA (v. 2.1b.4). rRNA-filtered and trimmed reads were aligned with STAR (v. 2.5.2b-0) against D. melanogaster genome release 6 (dm6). Coverage data were analyzed with CollectRnaSeqMetrics (v. 2.7.1.1) part of Picard tools, http://broadinstitute.github.io/picard/. Count tables were generated with featureCounts (v. 1.6.0.3). Read distribution among apical and basal samples was analyzed with Integrative Genomics Viewer (IGV) (v. 2.4.17). Statistical analysis was perfomed on R Studio (R 3.6.3) with Bioconductor v 3.10, with DEseq2 (differential gene expression analysis) (v 1.26.0), and rstatix (v 0.7.0) packages. Data visualization was performed with ggplot2 (v. 3.3.5) and ComplexHeatmap (v 2.1.1) R packages. Quantification of fluorescence signal was performed on Fiji (v. 2.0.0-rc-69/1.52i).

For manuscripts utilizing custom algorithms or software that are central to the research but not yet described in published literature, software must be made available to editors and reviewers. We strongly encourage code deposition in a community repository (e.g. GitHub). See the Nature Portfolio guidelines for submitting code & software for further information.

Data

Policy information about availability of data

All manuscripts must include a data availability statement. This statement should provide the following information, where applicable:

- Accession codes, unique identifiers, or web links for publicly available datasets
- A description of any restrictions on data availability
- For clinical datasets or third party data, please ensure that the statement adheres to our policy

The authors declare that all data supporting the findings of this study are available within the article and its supplementary information files. Raw microscopy images are available upon request. The D. melanogaster genome release 6 (dm6) data used in this study are available in the NCBI database under accession code GCA_000001215.4 (https://www.ncbi.nlm.nih.gov/assembly/GCF_000001215.4/). The raw RNA-seq data generated in this study have been deposited in the ArrayExpress database under accession code E-MTAB-9127 (https://www.ebi.ac.uk/arrayexpress/experiments/E-MTAB-9127/). The processed RNA-seq data are provided in the Supplementary Files (Supplementary Data 1). Source data are provided with this paper.

Human research participants

Policy information about studies involving human research participants and Sex and Gender in Research.

Reporting on sex and gender	N/A
Population characteristics	N/A
Recruitment	N/A
Ethics oversight	N/A

Note that full information on the approval of the study protocol must also be provided in the manuscript.

Field-specific reporting

Please select the one below that is the best fit for your research. If you are not sure, read the appropriate sections before making your selection.

☒ Life sciences ☐ Behavioural & social sciences ☐ Ecological, evolutionary & environmental sciences

For a reference copy of the document with all sections, see nature.com/documents/nr-reporting-summary-flat.pdf

Life sciences study design

All studies must disclose on these points even when the disclosure is negative.

Sample size	RNA-seq sample size and microdissected areas were determined on the basis of current related literature treating LCM samples (PMID: 28798045; PMID: 27387371; PMID: 28207000). Statistical analysis was performed on at least 3 different biological replicates (egg chambers from at least 3 different flies) to ensure reproducibility. We did not perform any statistical test to determine the sample size.
Data exclusions	RNA-seq replicates A5 (Apical replicate 5) and B5 (Basal replicate 5) were excluded from further analysis due to the high degree of dissimilarity with other apical and basal replicates, respectively. The degree of dissimilarity was evaluated by PCA analysis and Euclidean distance. This is explained in the Materials and Methods section.
Replication	All experiments were successfully replicated more than once on different days and in ovaries from different animals of each relevant genotype.
Randomization	Randomization was always performed whenever possible (smFISH validation; pharmacological treatment); in these cases, ovaries from different wild-type flies were randomly allocated in each experimental condition.
Blinding	Blinding was performed when analyzing different experimental conditions (RNAi, drug treatment). Complete blinding of smFISH probe names was not possible when mosaic tissues were analyzed due to the characteristic, thus recognizable, smFISH patterns of RNAs analyzed in this study in wt cells surrounding mutant cells. To avoid a subjective and qualitative interpretation of images, we have quantitatively analyzed all smFISH images with unbiased statistical methods.

Reporting for specific materials, systems and methods

We require information from authors about some types of materials, experimental systems and methods used in many studies. Here, indicate whether each material, system or method listed is relevant to your study. If you are not sure if a list item applies to your research, read the appropriate section before selecting a response.

Materials & experimental systems

n/a	Involved in the study
☐	☒ Antibodies
☒	☐ Eukaryotic cell lines
☒	☐ Palaeontology and archaeology
☐	☒ Animals and other organisms
☒	☐ Clinical data
☒	☐ Dual use research of concern

Methods

n/a	Involved in the study
☒	☐ ChIP-seq
☒	☐ Flow cytometry
☒	☐ MRI-based neuroimaging

Antibodies

Antibodies used

Antibodies used were:
- anti-Egl (gift from Ruth Lehmann, Whitehead Institute, Cambridge, MA, Rabbit) 1:1000
- anti-rabbit Alexa fluor 647 (Jackson ImmunoResearch Labs Cat# 111-605-003, RRID: AB_2338072, goat) 1:750

Validation

- anti-Egl was validated by Mach and Lehmann, 1997 (PMID: 9042857)
- anti-rabbit Alexa fluor 647 was referenced by PMID:28561736; PMID: 28592694; PMID: 29551269; PMID: 30015619; PMID: 30917329; PMID: 32343840; PMID: 32415988; and others.

Animals and other research organisms

Policy information about studies involving animals; ARRIVE guidelines recommended for reporting animal research, and Sex and Gender in Research

Laboratory animals

All organisms used in this study were female Drosophila melanogaster, well-fed and freshly eclosed (max 1-2 days old).

Lines used in this studies were as follows:

Commercial lines (Bloomington Drosophila Stock Center (BDSC)):
- w1118 (wild-type; #3605);
- DhcRNAi (#36698);
- eglRNAi (#28969);
- KhcRNAi (#35409);
- UAS-NLS-mCherry, (#38425);
-osk-Gal4 (#44242);
- VK33 (#9750);

Gifts from other labs:
- HsFLP; arm>f+>Gal4; UAS-CD8-mCherry (gift of Juan Manuel Gomez Elliff);
- tj-Gal4/CyO (gift of Juan Manuel Gomez Elliff);
- Tm1eg1/TM3Sb,Ser (Erdelyi et al., 1995)
- Tm1eg9/TM3Sb,Ser (Erdelyi et al., 1995)
- Dhc64C-GFP (Gaspar et al., 2021);
- GFP-Mago (Newmark et al., 1997);
-vasa-Gal4/TM3Sb (gift of Jean Rene Huynh);
- UAS-deltaC-Pym-GFP (Ghosh et al.,2014);
- UAS-Egl (Bullock et al., 2006);
- eglWU50/SM1 (Mach and Lehmann, 1997);
- eglPR29/SM6A (Mach and Lehmann, 1997);

Lines generated in this study (transgene sequences listed in Supplementary Data 2):
- UAS-GFP;
- UAS-0BicD-GFP;
- UAS-(+1)BicD-GFP;
- UAS-(-1)BicD-GFP.

Wild animals

The study did not involve wild animals.

Reporting on sex

Only female fruit flies have been used in the experiments, as the study investigated RNAs localizing in ovaries.

Field-collected samples

The study did not involve samples collected in the field.

Ethics oversight

No ethical approval was required for the Drosophila lines used.

Note that full information on the approval of the study protocol must also be provided in the manuscript.
